# A conservation checklist of the amphibians and reptiles of the State of Mexico, Mexico with comparisons with adjoining states

**DOI:** 10.3897/zookeys.953.50881

**Published:** 2020-07-27

**Authors:** Julio A. Lemos-Espinal, Geoffrey R. Smith

**Affiliations:** 1 Laboratorio de Ecología-UBIPRO, FES Iztacala UNAM, Avenida los Barrios 1, Los Reyes Iztacala, Tlalnepantla,edo. de México, 54090, México Universidad Nacional Autónoma de México Tlalnepantla Mexico; 2 Department of Biology, Denison University, Granville, Ohio 43023, USA Denison University Granville United States of America

**Keywords:** checklist, crocodilians, frogs, herpetofauna, lizards, salamanders, snakes, turtles

## Abstract

The State of Mexico has a unique combination of geographic characteristics and topography that promotes a high biodiversity. Unfortunately, continued human population growth of the metropolitan areas of Mexico City and Toluca have degraded the environment of the State of Mexico, which threatened its wildlife. An updated checklist of the amphibians and reptiles of the State of Mexico is provided and their conservation status summarized. The State of Mexico has 49 species of amphibians and 101 species of reptiles. The majority of the amphibians (73.5%) and reptiles (70.3%) found in the State of Mexico are endemic to Mexico. Of the amphibian and reptile species in the State of Mexico, 20.1% are IUCN listed (i.e., Vulnerable, Near Threatened, or Endangered), 18.4% are placed in a protected category by SEMARNAT (excluding NL and Pr, this last category is equivalent to the LC category of IUCN), and 34.9% are categorized as high risk by the EVS. The importance of forested habitats for the protected amphibians and reptiles in the State of Mexico suggest that management of these habitats to maintain or expand them needs to be considered.

## Introduction

Although relatively small, the State of Mexico bears unique geographic characteristics that combined with its topography create conditions that promote a high level of biodiversity. Unfortunately, these same conditions along with the continued human population growth of the metropolitan area of Mexico City and the city of Toluca have created high water and air pollution levels, deforestation, habitat fragmentation, and low water availability, which threaten the wildlife of this state (Rodríguez Romero et al. 2008; Flores-Villela et al. 2010). For example, atmospheric water in the Valley of Mexico contains heavy metals that are detectable and exceed regulatory limits when condensed (Bautista-Olivas et al. 2014). This is especially important for amphibians and reptiles, which are represented in the State of Mexico by a unique assortment of species. Central Mexico, including the State of Mexico, contains several areas of high endemicity for the herpetofauna of Mexico and as such is very important to the conservation of the Mexican herpetofauna (Flores-Villela et al. 2010).

Here, we provide an updated checklist of the amphibians and reptiles documented in the State of Mexico. We also summarize the conservation status of these species with the goal of determining if there are particular taxa of conservative concern in the State of Mexico. In addition, we consider the overlap in species between the State of Mexico and its neighboring states.

### Physiographic characteristics of the state

**Figure 1.**
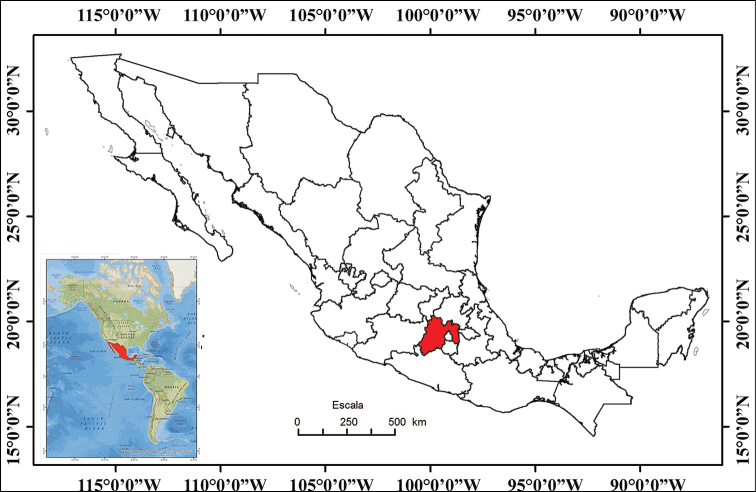
Map of Mexico with the State of Mexico shown in red (modified from INEGI, 2018a).

The State of Mexico is the most populous, as well as the most densely populated state in Mexico. It is located in south-central Mexico, in the highest part of the Mexican Altiplano, between 18°22'0.84"N and 20°17'9.24"N, and 100°36'46.8"W and 98°35'48.84"W (Fig. 1). It is bordered by the states of Querétaro and Hidalgo to the north, Morelos and Guerrero to the south, Michoacán to the west, Tlaxcala and Puebla to the east, and surrounds Mexico City on three sides (west, north, and east). The state is relatively small (22,351 km^2^) and is the seventh smallest Mexican state, representing 1% of the total surface territory of Mexico (modified from Wikipedia – https://en.wikipedia.org/wiki/State_of_Mexico – accessed 21 November 2019).

**Figure 2.**
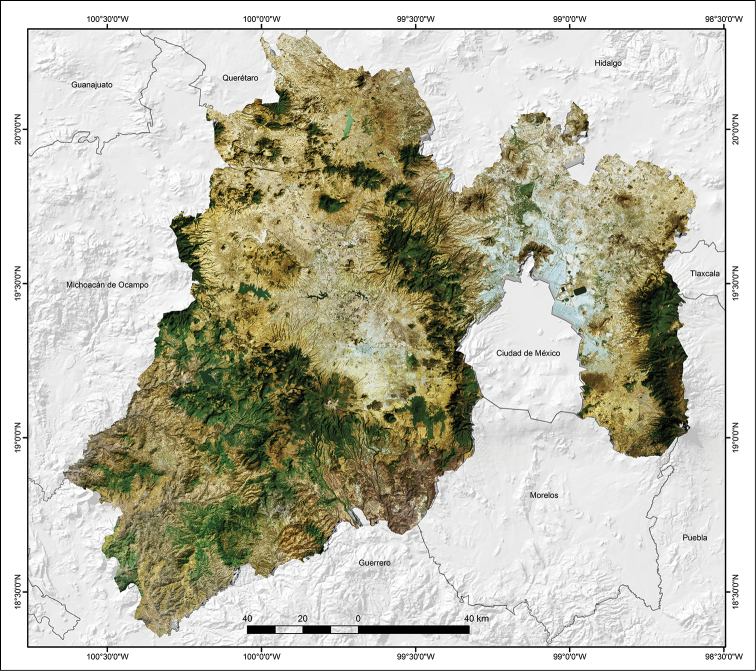
Topographical map of the State of Mexico, Mexico (CONABIO, 1997).

**Figure 3.**
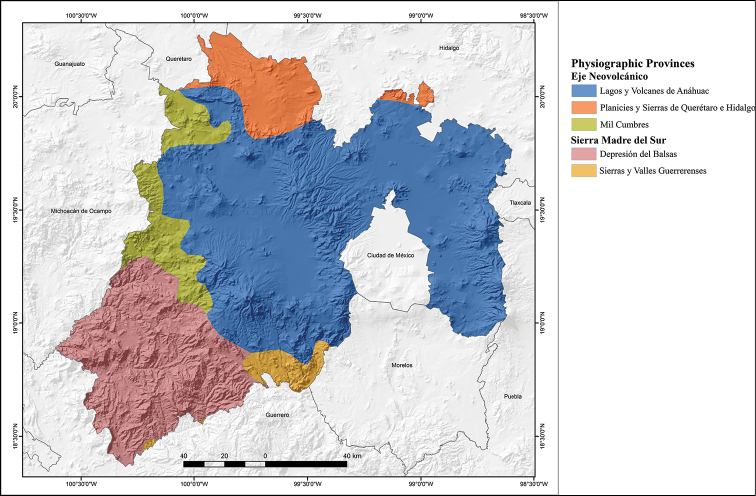
Physiographic provinces of the State of Mexico, Mexico (modified from Cervantes-Zamora et al. 1990).

The topography of the state is highly variable, with the highest mountains in the extreme eastern part of the state along the border with Puebla (Popocatépetl 5,380 m altitude, Iztaccíhuatl 5,203 m, Monte Tláloc 4,120 m), and in the central part of the state (Nevado de Toluca 4,643 m), as well as rugged intermontane valleys, hills and plains, with altitudes ranging from 300 m near the border with Guerrero to 5,380 m on the top of the Popocatépetl Volcano (Fig. 2). The State of Mexico contains two physiographic provinces: a) Eje Neovolcánico, and b) Sierra Madre del Sur (Fig. 3; modified from INEGI 2017). The Eje Neovolcánico comprises most of the state, occupying the central, northern and eastern portions of the state. This province is divided into three sub-provinces: a) Lagos y Volcanes de Anáhuac, which occupies most of the central, north, and east portions of the state, and includes the northern part of the Metropolitan Zone of Mexico and the city of Toluca. b) Mil Cumbres, a thin strip running from north to south and lying between the sub-provinces of Lagos y Volcanes de Anáhuac and Depresión del Balsas, and eastern Michoacán. c) Planicies y Sierras de Querétaro e Hidalgo, a small portion at the northern end of the state that borders Querétaro and Hidalgo. The Sierra Madre del Sur comprises the southwestern corner of the state along its border with Guerrero and western-northwestern Morelos, and is divided into two sub-provinces: a) Depresión del Balsas, which is bordered by northern Guerrero, and b) Sierras y Valles Guerrerenses, which is a small area bordering northern Guerrero and western-northwestern Morelos (Fig. 3).

**Figure 4.**
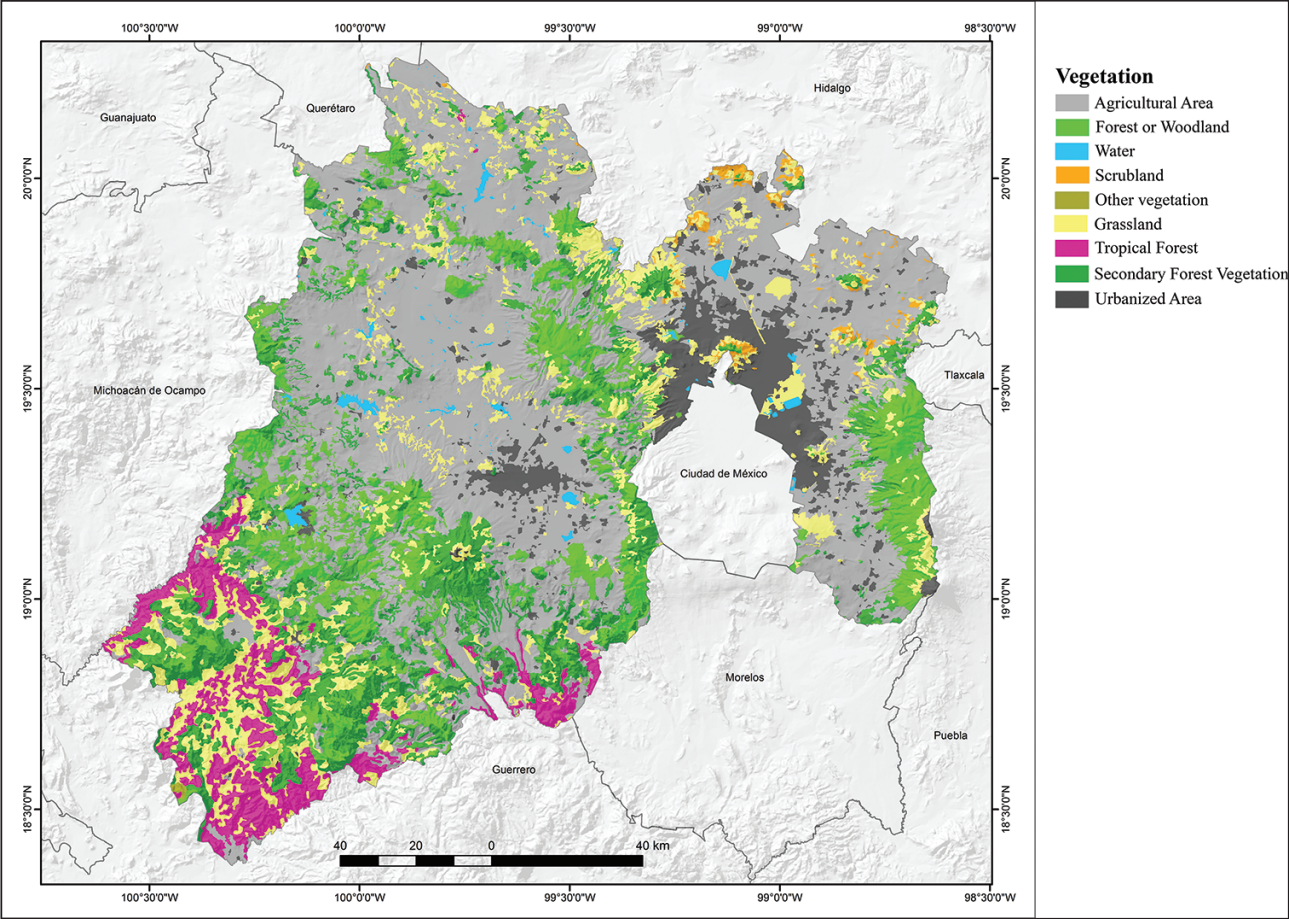
Vegetation map of the State of Mexico, Mexico (modified from Dirección General de Geografía – INEGI 2016).

The State of Mexico has a variety of vegetation types (Fig. 4; modified from INEGI 2017). Agricultural Areas that occupy 54.61% of the state’s surface area, and are found mainly in the central, northern, and eastern parts of the state, occupying most of the province of the Eje Neovolcánico. Woodlands cover 27.22% of the state’s surface area, and are scattered at the higher elevations of the Eje Neovolcánico province, especially the western foothills of the Popocatépetl and Iztaccíhuatl volcanoes, the Sierra de las Cruces – Sierra del Ajusco complex, the area surrounding the Nevado de Toluca Volcano, and most of the Mil Cumbres Subprovince. Woodlands include Oak Forests which are distributed between 1,600 and 2,400 meters above sea level; Pine-Oak Forest, which develops above 2,400 meters altitude; and Pine Forest, which develops in the highest elevations of the state’s mountains. At the highest elevations, this forest is surrounded by padded grasses including *Mülhenbergia rigida*, *Stipa ichu*, and *Bouteloa gracilis* among others. Grasslands, covering 12.15% of state’s surface area, occur in isolated areas in the northern, central, and southwestern parts of the state and they intermingle with Tropical Forests, which are limited to some scattered spots in the Subprovinces of the Sierra Madre del Sur. Tropical Forest, comprising 5.34% of the state’s surface area, is represented by Tropical Deciduous Forest, also called Tropical Dry Forest, that develops between 1,500 and 1,600 m altitude. These forests, although lush, lose their leaves during the dry season (winter-spring), and have dense foliage during the rainy season (summer). Scrubland covers only 0.2% of the state’s surface area. The remaining 0.41% is represented by scattered areas lacking vegetation (Fig. 4).

**Figure 5.**
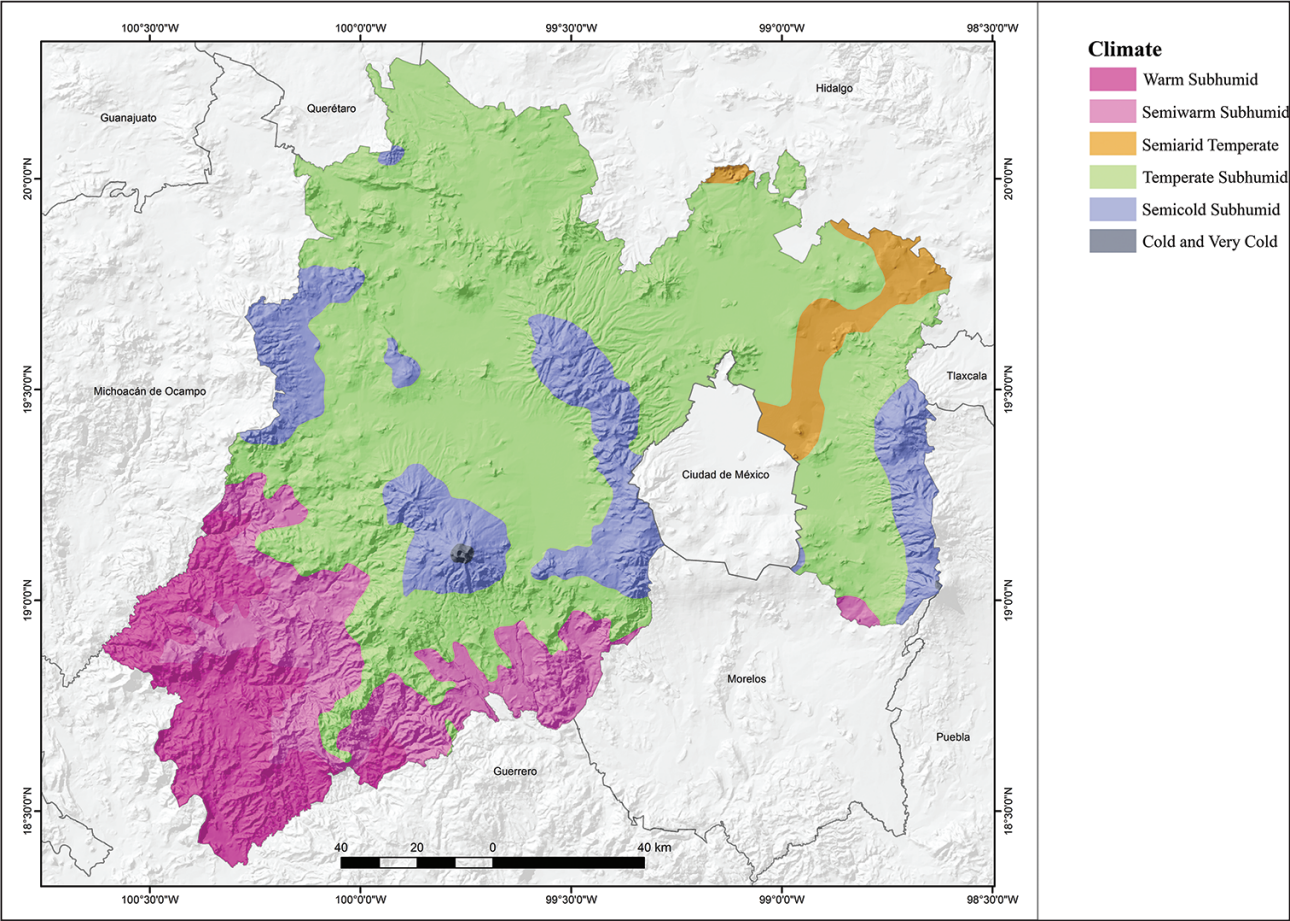
Climate map of the State of Mexico, Mexico (modified from García – Comisión Nacional para el Conocimiento y Uso de la Biodiversidad 1998).

Given the geographical location and diversity of the natural regions in the state, there are several climates in the State of Mexico (Fig. 5; modified from López-Cano et al. 2009; INEGI 2017). A warm sub-humid climate with summer rains and semi-humid with summer rains is found in the Balsas Basin in the extreme southwestern part of the state, covering 20.8% of the state area. The temperate sub-humid with summer rains is found over most of the Lerma Basin and Valley of Mexico, covering most of the state (61.7% of the state). The wet semi-cold climate with abundant rains in summer and sub-humid semi-cold with summer rains is present in the highest mountains of the state (Nevado de Toluca, Sierra Nevada, Sierra las Cruces, Sierra del Ajusco, etc.), covering 11.6% of the state surface. The temperate semi-dry climate is found in the northeast corner of the state, in a strip that runs from the central eastern part of the state, on the northeastern limit of Mexico City to northeastern State of Mexico on the border with Hidalgo, covering 5.7% of the state surface. A Cold climate present on the summits of the Nevado de Toluca, Popocatépetl, and Iztaccíhuatl volcanoes, covering 0.2% of the state’s area.

## Materials and methods

We compiled our list of amphibians and reptiles of the State of Mexico from our field work over several years, especially within the past 5–10 years, a thorough examination of available literature on amphibians and reptiles in the state, amphibian and reptile records for the State of Mexico in VertNet.org, and amphibian and reptile records for the State of Mexico in Servicio de Descarga de Ejemplares del Sistema Nacional de Información sobre Biodiversidad (SNIB-CONABIO), data bases Amphibians State of Mexico and Reptiles State of Mexico. Amphibian names follow Frost (2019) and AmphibiaWeb (2019) (http://amphibiaweb.org) and reptile names follow Uetz and Hošek (2019). We included species in the list only if we could confirm records by either direct observation or documented museum records or vouchers.

We made species accumulation curves for the total herpetofauna, and amphibians and reptiles separately using the year of the first recorded observation for each species. These curves can estimate the potential species richness of amphibians and reptiles (see Raxworthy et al. 2012). For each species, we recorded conservation status based on the IUCN Red List 2019-2, listing in SEMARNAT (2019), and Environmental Vulnerability Scores (Wilson et al. 2013a, b; Johnson et al. 2015). We determined the number of species found in the State of Mexico that overlapped with neighboring states and Mexico City using recent state lists (Michoacán, Alvarado-Díaz et al. 2013; Hidalgo, Lemos-Espinal and Smith 2015; Puebla, Woolrich-Piña et al. 2017; Guerrero, Palacios-Aguilar and Flores-Villela 2018; Mexico City, Lemos-Espinal and Smith in press; Morelos, Lemos-Espinal and Smith 2020; and Querétaro, Cruz-Elizalde et al. 2019). We did not include the state of Tlaxcala since no comprehensive check list of the amphibians and reptiles of this state currently exists. We generated border lengths with the INEGI state division map for the year 2018 using ArcMap 10.7.1 neighboring polygon tool (June 2019).

## Results and discussion

**Figure 6.**
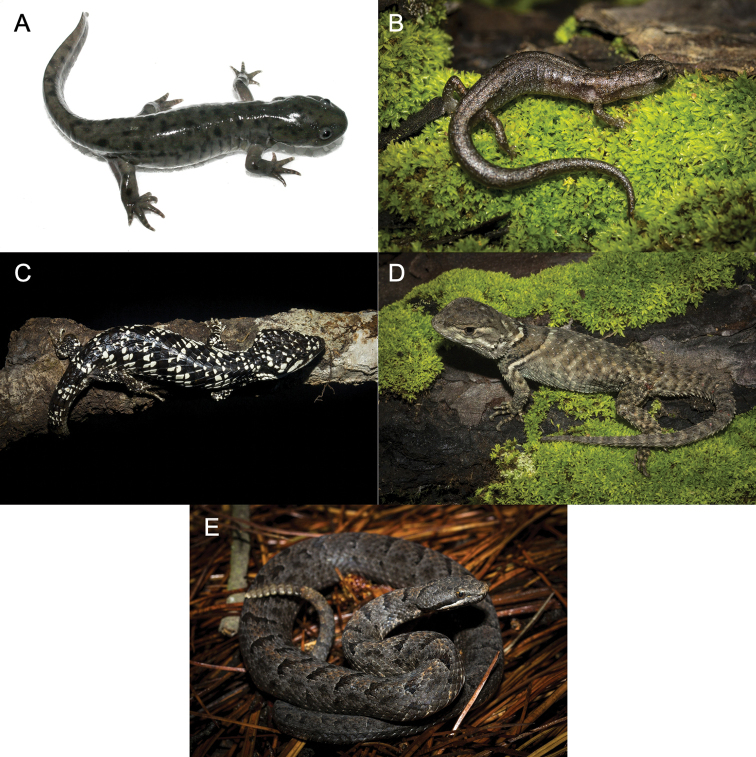
**A***Ambystoma lermaense***B***Chiropterotriton orculus***C***Abronia deppii***D** juvenile *Sceloporus sugillatus***E***Crotalus transversus*. Photos by Eric Centenero-Alcalá

The State of Mexico is home to 150 species of amphibians and reptiles representing 31 families (two introduced: Gekkonidae and Typhlopidae) and 65 genera (two introduced: *Hemidactylus* and *Indotyphlops*) (Table 1; Fig. 6). The herpetofauna of the State of Mexico includes 49 species of amphibians (33 anurans [one introduced], and 16 salamanders), and 101 reptiles (40 lizards [one introduced], 57 snakes [one introduced], and four turtles). The three introduced species are the American Bullfrog (*Rana catesbeiana*), the Common House Gecko (*Hemidactylus frenatus*), and the Brahminy Blindsnake (*Indotyphlops braminus*). Five of the 147 native species of the State of Mexico are endemic to the state: the Delicate-skinned Salamander (*Ambystoma bombypellum*), the Granular Salamander (*Ambystoma granulosum*), the Lake Lerma Salamander (*Ambystoma lermaense*), Roberts’ False Brook Salamander (*Pseudoeurycea robertsi*), and the Herrera Alligator Lizard (*Barisia herrerae*). The most species rich families of amphibians in the State of Mexico are Hylidae, Ambystomatidae, and Plethodontidae, whereas the most species rich families of reptiles are Phrynosomatidae and Colubridae (Table 1).

**Figure 7.**
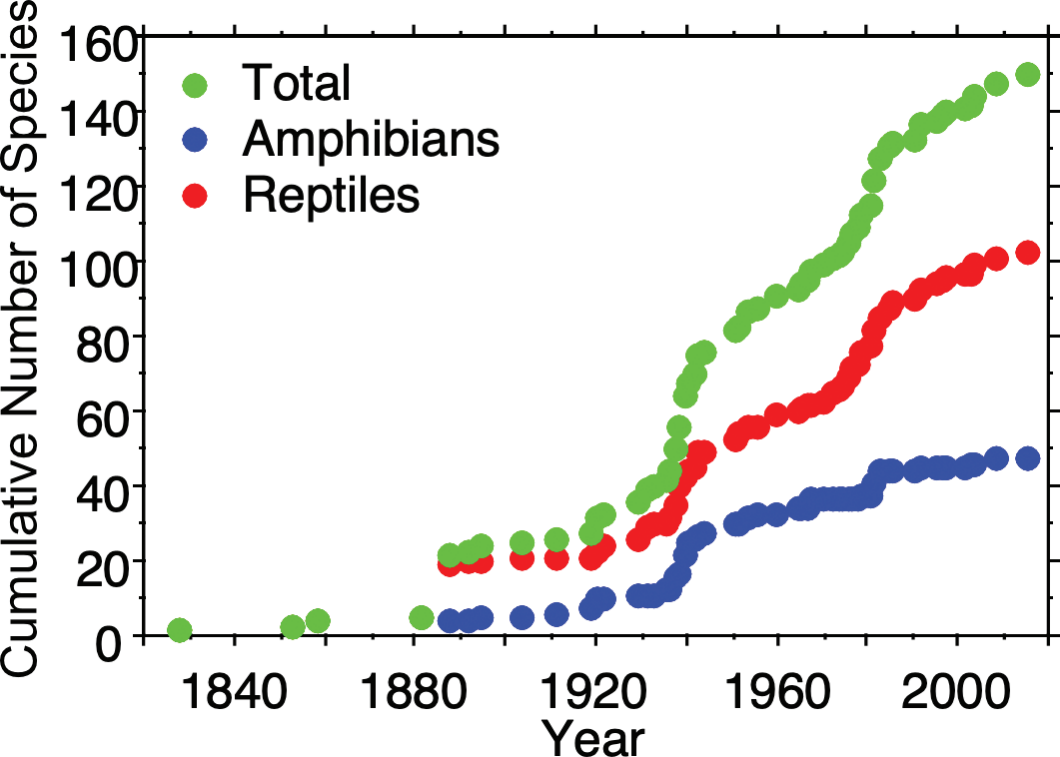
Species accumulation curves for total herpetofauna, amphibians, and reptiles of the State of Mexico, Mexico.

The species accumulation curves for the total herpetofauna, reptiles, and amphibians all show a steep increase in the number of species documented in the State of Mexico in the second half of the 20^th^ century, and that trend appears to be continuing, albeit at a somewhat slower rate in the 21^st^ century (Fig. 7). This suggests that the overall number of amphibians and reptiles in the State of Mexico is likely to increase over time. Indeed, we compiled a list of 21 species (two amphibians, 19 reptiles: Table 2) that potentially occur in the State of Mexico (Table 2). These potential species are distributed mainly along the border with Guerrero (extreme southwestern State of Mexico), Hidalgo and Querétaro (northern State of Mexico), Morelos (southern State of Mexico), and Puebla (eastern State of Mexico), and are based on distributional records appearing in Vertnet.org, the Sistema Nacional de Información sobre Biodiversidad (SNIB-CONABIO) for all six neighboring states and Mexico City, Dixon and Lemos-Espinal (2010) for Querétaro; and Lemos-Espinal and Dixon (2016) for Hidalgo. We are convinced that as more herpetological work is done in the areas near the borders between the State of Mexico and its neighboring states, these potential species will likely be documented in the State of Mexico.

### General distribution

Thirty-six of the 49 species of amphibians found in the State of Mexico are endemic to Mexico, four of them to the State of Mexico (*Ambystoma bombypellum*, *A. granulosum*, *A. lermaense*, and *Pseudoeurycea robertsi*); twelve are species found mainly along the Eje Neovolcánico of central Mexico; seven are species typical of the Pacific Coast, including the Balsas Depression; three are species characteristics of the Mexican Plateau; seven more are species with a widely distributional patterns in the Mexican Plateau, the Sierra Madre Occidental, Sierra Madre Oriental, Sierra Madre del Sur, and Eje Neovolcánico; and the remaining three are represented by scattered populations in the Mexican Plateau, Sierras Madres, and Eje Neovolcánico (Table 1). Of the 13 amphibian species not endemic to Mexico, four are found in the United States and Mexico, five range from Mexico to Central or even South America, three more are found from southern United States to Central or South America, and one is introduced (Table 1). Thirty-three of the 40 species of lizards that occur in the state are endemic to Mexico; one is endemic to the State of Mexico (*Barisia herrerae*); six are restricted to localities in central Mexico in the State of Mexico, Morelos, Puebla, and Mexico City; ten are typical of the Mexican Pacific Coast; two are limited to the Eje Neovolcánico of central Mexico; six are limited to the central-south part of Mexico, in the Eje Neovolcánico and Sierra Madre del Sur; two are typical of the Mexican Plateau, occurring also in the Eje Neovolcánico or the Sierra Madre del Sur; and six occur in both the Sierra Madre Occidental and Sierra Madre Oriental, and in the Eje Neovolcánico. Of the seven species of lizards found in the State of Mexico but that are not endemic to Mexico, one is found in the United States and Mexico, four are distributed from Mexico to Central America, one is distributed from the United States to Central America, and one is introduced (Table 1). Thirty-six of the 57 species of snakes that inhabit the State of Mexico are endemic to Mexico. Of the 21 snake species not endemic to Mexico that are found in the State of Mexico, six are found in the United States and Mexico, ten range from Mexico to Central or even South America, four are found from central or southern United States to Central or South America, and one is introduced (Table 1). Two of the four species of turtles found in the State of Mexico are endemic to Mexico, one is a species found in the United States and Mexico, and one is distributed from Mexico to Central America (Table 1).

**Table 1.** Amphibians and reptiles of the State of Mexico with distributional and conservation status. Vegetation Type: (1 = Oak Forest; 2 = Pine-oak Forest; 3 = Pine Forest; 4 = Tropical Deciduous Forest; 5 = Grassland; 6 = Scrubland); IUCN Status: (DD = Data Deficient; LC = Least Concern, VU = Vulnerable, NT = Near Threatened; EN = Endangered; CR = Critically Endangered; NE = not Evaluated) according to the IUCN Red List (IUCN 2019); Environmental Vulnerability Score: (EVS – the higher the score the greater the vulnerability: low (L) vulnerability species (EVS of 3–9); medium (M) vulnerability species (EVS of 10–13); and high (H) vulnerability species (EVS of 14–20) (Wilson et al. 2013a,b; Johnson et al. 2015); conservation status in Mexico according to SEMARNAT (2019): (P = in danger of extinction, A = threatened, Pr = subject to special protection, NL – not listed). Global Distribution: 0 = Endemic to the State of Mexico; 1 = Endemic to Mexico; 2 = Shared between the US and Mexico; 3 = widely distributed from Mexico to Central or South America; 4 = widely distributed from the US to Central or South America; IN = Introduced to State of Mexico. Date in which the first record appeared; and Source of the first record.

	Vegetation type	IUCN status	SEMARNAT	EVS	Global distribution	Year of first record	Source
**CLASS AMPHIBIA**							
**ORDER ANURA**							
**FAMILY BUFONIDAE (5)**							
*Anaxyrus compactilis* (Wiegmann, 1833)	1,2,5,6	LC	NL	H (14)	1	1888	Dugès 1888
*Incilius marmoreus* (Wiegmann, 1833)	4	LC	NL	M (13)	1	1930	MCZ-A 17755
*Incilius occidentalis* (Camerano, 1879)	1,2,3,6	LC	NL	M (11)	1	1941	TCWC 6365
*Incilius perplexus* (Taylor, 1943)	4	EN	NL	M (11)	1	1983	Camarillo-Rangel 1983
*Rhinella horribilis* (Wiegmann, 1833)	4	LC	NL	L (3)	4	1941	UIMNH 25155
**FAMILY CRAUGASTORIDAE (4)**							
*Craugastor augusti* (Dugès, 1879)	2,6	LC	NL	L (8)	2	1942	Taylor 1942
*Craugastor hobartsmithi* (Taylor, 1937)	2	EN	NL	H (15)	1	1936	UIMNH 18301
*Craugastor pygmaeus* (Taylor, 1937)	1,2,3	VU	NL	L (9)	3	1992	Camarillo-Rangel and Smith 1992
*Craugastor rugulosus* (Cope, 1870)	3	LC	NL	M (13)	3	1968	UTEP Herp:7475
**FAMILY ELEUTHERODACTYLIDAE (4)**							
*Eleutherodactylus angustidigitorum* (Taylor, 1940)	1,2,3,6	VU	Pr	H (17)	1	1954	TCWC 11158
*Eleutherodactylus maurus* Hedges, 1989	1,2,3	DD	Pr	H (17)	1	1954	TCWC 11259
*Eleutherodactylus nitidus* (Peters, 1870)	1,2,3	LC	NL	M(12)	1	1951	AMNH A-55227
*Eleutherodactylus pipilans* (Taylor, 1940)	4	LC	NL	M (11)	3	1979	MZFC 3764
**FAMILY HYLIDAE (9)**							
*Dryophytes arenicolor* (Cope, 1886)	1,2,3,4,5,6	LC	NL	L (7)	2	1921	MCZ A-8367
*Dryophytes eximius* (Baird, 1854)	1,2,3,6	LC	NL	M (10)	1	1919	AMNH A 13256
*Dryophytes plicatus* (Brocchi, 1877)	1,2,3,6	LC	A	M (11)	1	1912	MCZ-A 25699
*Exerodonta smaragdina* (Taylor, 1940)	4	LC	Pr	M (12)	1	1992	Camarillo-Rangel and Smith 1992
*Sarcohyla bistincta* (Cope, 1877)	1,2,3	LC	Pr	L (9)	1	1938	UIMNH 17903
*Sarcohyla pentheter* (Adler, 1965)	4	EN	NL	M (13)	1	2009	Aguilar-Miguel et al. 2009
*Smilisca baudinii* (Duméril & Bibron, 1841)	1,2,4,6	LC	NL	L (3)	4	1982	CNAR 3912
*Smilisca fodiens* (Boulenger, 1882)	4	LC	NL	L (8)	2	1968	UTEP H 8448
*Tlalocohyla smithii* (Boulenger, 1902)	4	LC	NL	M (11)	1	1968	UTEP H 7713
**FAMILY LEPTODACTYLIDAE (1)**							
*Leptodactylus melanonotus* (Hallowell, 1861)	4	LC	NL	L (6)	3	1965	ENCB 7687
**FAMILY MICROHYLIDAE (1)**							
*Hypopachus variolosus* (Cope, 1866)	4	LC	NL	L (4)	4	1941	ENCB 2905
**FAMILY PHYLLOMEDUSIDAE (1)**							
*Agalychnis dacnicolor* (Cope, 1864)	4	LC	NL	M (13)	1	1983	Camarillo-Rangel 1983
**FAMILY RANIDAE (7)**							
*Rana catesbeiana* Shaw, 1802	**IN**	**IN**	**IN**	**IN**	**IN**	1982	CNAR 17313
*Rana forreri* Boulenger, 1883	4	LC	Pr	L (3)	3	1940	CNAR 620
*Rana montezumae* Baird, 1854	1,2,3,5,6	LC	Pr	M (13)	1	1888	Dugès 1888
*Rana neovolcanica* Hillis & Frost, 1985	1,2,3	NT	A	M (13)	1	2009	MZFC 23392
*Rana spectabilis* Hillis & Frost, 1985	1,2,3,5,6	LC	NL	M (12)	1	1936	FMNH 110654
*Rana tlaloci* Hillis & Frost, 1985	1,2,5,6	CR	P	H (15)	1	1979	ENCB 10567
*Rana zweifeli* Hillis, Frost & Webb, 1984	1,2,3,4,5	LC	NL	M (11)	1	1982	ENCB 11912
**FAMILY SCAPHIOPODIDAE (1)**							
*Spea multiplicata* (Cope, 1863)	1,5	LC	NL	L (3)	2	1940	UIMNH 27893
**ORDER CAUDATA**							
**FAMILY AMBYSTOMATIDAE (8)**							
*Ambystoma altamirani* Dugès, 1895	1,2,3,5	EN	A	M (13)	1	1895	Dugès 1895
*Ambystoma bombypellum* Taylor, 1940	2,5	CR	Pr	H (15)	0	1940	Taylor 1940a
*Ambystoma granulosum* Taylor, 1944	1,2,3,5	CR	Pr	H (14)	0	1944	Taylor 1944
*Ambystoma leorae* (Taylor, 1943)	2,3,5	CR	A	H (15)	1	1943	Taylor 1943
*Ambystoma lermaense* (Taylor, 1940)	5	EN	Pr	H (15)	0	1940	Taylor 1940a
*Ambystoma ordinarium* Taylor, 1940	?	EN	Pr	M (13)	1	22004	Matias-Ferrer and Murillo 2004a
*Ambystoma rivulare* (Taylor, 1940)	1,2,3,5	DD	A	M (13)	1	1940	Taylor 1940b
*Ambystoma velasci* Duges, 1888	1,2,3,5,6	LC	Pr	M (10)	1	1888	Dugès 1888
**FAMILY PLETHODONTIDAE (8)**							
*Aquiloeurycea cephalica* (Cope, 1865)	1,2,3,5	NT	A	H (14)	1	1938	UIMNH 30898
*Chiropterotriton orculus* (cope, 1865)	1,2,3,5	VU	NL	H (18)	1	1951	MVZ 54646
*Isthmura belli* (Gray, 1850)	1,2,3,5	VU	A	M (12)	1	1938	UIMNH 30881
*Pseudoeurycea altamontana* (Taylor, 1939)	1,2,3,5	EN	Pr	H (17)	1	1956	UCM 8117
*Pseudoeurycea leprosa* (Cope, 1869)	1,2,3,5	LC	A	H (16)	1	1921	UMMZ 56989
*Pseudoeurycea longicauda* Lynch, Wake, & Yang, 1983	1,2,3,5	EN	Pr	H (17)	1	1983	Lynch et al. 1983
*Pseudoeurycea robertsi* (Taylor, 1939)	1,2,3,5	CR	A	H (18)	1	1939	Taylor 1939
*Pseudoeurycea tlilicxitl* Lara-Góngora, 2003	1,2,3,5	EN	NL	H (17)	1	2003	Lara-Góngora 2003
**CLASS REPTILIA**							
**SUBORDER LACERTILIA**							
**FAMILY ANGUIDAE (5)**							
*Abronia deppii* (Wiegmann, 1828)	2	EN	A	H (16)	1	1979	MZFC 6294
*Barisia herrerae* Zaldivar-Riverón & Nieto Montes de Oca, 2002	2,3	EN	NL	H (15)	0	2002	Zaldivar-Riverón and Nieto Montes de Oca 2002
*Barisia imbricata* (Wiegmann, 1828)	1,2,3,5,6	LC	Pr	H (14)	1	1888	Dugès 1888
*Barisia rudicollis* (Wiegmann, 1828)	1,2,3,5	EN	P	H (15)	1	1828	Wiegmann 1828
*Gerrhonotus liocephalus* Wiegmann, 1828	1,2,3,5	LC	Pr	L (6)	1	1938	FMNH 112024
**FAMILY DACTYLOIDAE (1)**							
*Anolis nebulosus* (Wiegmann, 1834)	1,2,4	LC	NL	M (13)	1	1940	UCM 46440
**FAMILY GECKONIDAE (1)**							
*Hemidactylus frenatus* Duméril & Bribon, 1836	**IN**	**IN**	**IN**	**IN**	**IN**	1998	Casas-Andreu et al. 1998
**FAMILY HELODERMATIDAE (1)**							
*Heloderma horridum* (Wiegmann, 1829)	4	LC	A	M (11)	3	1933	MVZ Herp 16434
**FAMILY IGUANIDAE (1)**							
*Ctenosaura pectinata* (Wiegmann, 1834)	4	NE	A	H (15)	1	1982	CNAR 3910
**FAMILIY PHRYNOSOMATIDAE (19)**							
*Phrynosoma orbiculare* (Linnaeus, 1758)	1,2,5,6	LC	A	M (12)	1	1888	Dugès 1888
*Sceloporus aeneus* Wiegmann, 1828	5,6	LC	NL	M (13)	1	1921	MCZ R-16069
*Sceloporus anahuacus* Lara-Góngora, 1983	1,2,3	LC	NL	H (15)	1	1979	UCM 52300
*Sceloporus bicanthalis* Smith, 1937	5	LC	NL	M (13)	1	1937	MCZ R-170033
*Sceloporus dugesii* Bocourt, 1874	2,5	LC	NL	M (13)	1	1983	CNAR 5006
*Sceloporus gadoviae* Boulenger, 1905	4	LC	NL	M (11)	1	1996	CNAR 12304
*Sceloporus grammicus* Wiegmann, 1828	1,2,3,6	LC	Pr	L (9)	1	1888	Dugès 1888
*Sceloporus horridus* Wiegmann, 1834	4	LC	NL	M (11)	1	1951	AMNH R-71351
*Sceloporus megalepidurus* Smith, 1934	4	VU	Pr	H (14)	1	1971	MCZ R-133166
*Sceloporus melanorhinus* Bocourt, 1876	4	LC	NL	L (9)	3	1977	MZFC 6312
*Sceloporus mucronatus* Cope, 1885	2,3,5	LC	NL	M (13)	1	1939	USNM 112207
*Sceloporus ochoterenae* Smith, 1934	4	LC	NL	M (12)	1	1992	Camarillo-Rangel and Smith 1992
*Sceloporus palaciosi* Lara-Góngora, 1983	1,2,3	LC	NL	H (15)	1	1976	USNM 245337
*Sceloporus pyrocephalus* Cope, 1864	4	LC	NL	M (12)	1	1982	CNAR 3900
*Scelopours scalaris* Wiegmann, 1828	5,6	LC	NL	M (12)	1	1888	Dugès 1888
*Sceloporus spinosus* Wiegmann, 1828	1,2,5,6	LC	NL	M (12)	1	1922	MVZ 8851
*Sceloporus sugillatus* Smith, 1942	1,2,3	LC	NL	H (16)	1	1939	UIMNH 10753
*Sceloporus torquatus* Wiegmann, 1828	1,2,3,5,6	LC	NL	M (11)	1	1888	Dugès 1888
*Urosaurus bicarinatus* (Duméril, 1856)	4	LC	NL	M (12)	1	1930	MCZ R-33686
**FAMILY PHYLLODACTYLIDAE (1)**							
*Phyllodactylus lanei* Smith, 1935	4	LC	NL	H (15)	1	1981	CNAR 3550
**FAMILY SCINCIDAE (6)**							
*Marisora brachypoda* (Taylor, 1956)	4	LC	NL	L (6)	3	1882	USNM 12718
*Plestiodon brevirostris* (Günther, 1860)	1,2,3	LC	NL	M (11)	1	1942	KUNHM 25937
*Plestiodon copei* (Taylor, 1933)	1,2,3	LC	Pr	H (14)	1	1932	USNM 92547
*Plestiodon dugesii* (Thominot, 1883)	1,2,3	VU	Pr	H (16)	1	1954	KUNHM 38080
*Plestiodon indubitus* (Taylor, 1933)	1,2,3	NE	NL	H (15)	1	1932	UIMNH 22701
*Plestiodon lynxe* (Wiegmann, 1834)	1,2,3	LC	Pr	M (10)	1	1974	UTA 4182
**FAMILY TEIIDAE (5)**							
*Aspidoscelis communis* (Cope, 1878)	4	LC	Pr	H (14)	1	2009	Aguilar-Miguel et al. 2009
*Aspidoscelis costatus* (Cope, 1878)	4	LC	Pr	M (11)	1	1941	ENCB 6757
*Aspidoscelis deppii* (Wiegmann, 1834)	4	LC	NL	L (8)	3	1977	MZFC 5884
*Aspidoscelis gularis* (Baird & Girard, 1852)	4	LC	NL	L (9)	4	1930	MCZ Herp R-33685
*Aspidoscelis sackii* (Wiegmann, 1834)	4	LC	NL	H (14)	1	1966	ENCB 4285
**SUBORDER SERPENTES**							
**FAMILY BOIDAE (1)**							
*Boa sigma* Smith, 1943	4	NE	NL	H (15)	1	1985	Camarillo-Rangel et al. 1985
**FAMILY COLUBRIDAE (21)**							
*Conopsis biserialis* (Taylor & Smith, 1942)	1,2,3,6	LC	A	M (13)	1	1932	Taylor and Smith 1942
*Conopsis lineata* (Kennicott, 1859)	1,2,3,6	LC	NL	M (13)	1	1859	Kennicott 1859
*Conopsis nasus* (Günther, 1858)	1,2,3 ,6	LC	NL	M (11)	1	1921	MCZ R-16128
*Drymarchon melanurus* (Duméril, Bibron & Duméril, 1854)	4	LC	NL	L (6)	3	1975	ENCB 9028
*Drymobius margaritiferus* (Schlegel, 1837)	4	LC	NL	L (6)	3	1939	MCZ R-45575
*Lampropeltis polyzona* Cope, 1860	1,2,3,4,5	LC	NL	L (7)	1	1943	ENCB 2205
*Leptophis diplotropis* (Günther, 1872)	4	LC	A	H (14)	1	1978	CNAR 3264
*Masticophis mentovarius* (Duméril, Bibron & Duméril, 1854)	4	LC	A	L (6)	3	1960	KUNHM 67691
*Oxybelis aeneus* (Wagler, 1824)	4	LC	NL	L (5)	4	1985	Camarillo-Rangel et al. 1985
*Pituophis deppei* (Dumeril, 1853)	1,2,3,4,6	LC	A	H (14)	1	1853	Dumeril 1853
*Pituophis lineaticollis* (Cope, 1861)	1,2,3,4,5	LC	NL	L (8)	3	1940	UIMNH 36223 reported by Duellman 1960
*Pseudoficimia frontalis* (Cope, 1864)	4	LC	NL	M (13)	1	1951	AMNH R-71359
*Salvadora bairdi* Jan & Sordelli, 1860	1,2,3,4,5,6	LC	Pr	H (15)	1	1888	Dugès 1888
*Salvadora mexicana* (Duméril, Bibron & Duméril, 1854)	4	LC	Pr	H (15)	1	1982	CNAR 3908
*Senticolis triaspis* (Cope, 1866)	1,2,4,5	LC	NL	L (6)	3	1943	ENCB 2207
*Tantilla bocourti* (Günther, 1895)	1,2,5	LC	NL	L (9)	1	1960	KUNHM 67723
*Tantilla calamarina* Cope, 1866	4	LC	Pr	M (12)	1	1981	UTEP H-13999
*Tantilla deppei* (Bocourt, 1883)	4	LC	A	M (13)	1	1977	CNAR 1751
*Tantilla rubra* Cope, 1875	1,2,3,5	LC	Pr	L (5)	3	2009	Aguilar-Miguel et al. 2009
*Trimorphodon biscutatus* (Duméril, Bibron & Duméril, 1854)	4	NE	NL	L (7)	3	1983	Camarillo-Rangel 1983
*Trimorphodon tau* Cope, 1870	4	LC	NL	M (13)	1	1943	ENCB 2206
**FAMILY DIPSADIDAE (12)**							
*Conophis vittatus* Peters, 1860	4	LC	NL	M (11)	3	2004	Matias-Ferrer and Murillo 2004c
*Diadophis punctatus* (Linnaeus, 1766)	1,2,3,6	LC	NL	L (4)	2	1937	MZFC 2307
*Enulius flavitorques* (Cope, 1868)	4	LC	NL	L (5)	3	1951	AMNH R-71357
*Geophis bicolor* Günther, 1868	4	DD	Pr	H (15)	1	1992	Camarillo-Rangel and Smith 1992
*Geophis sieboldi* (Jan, 1862)	4	DD	Pr	M (13)	1	1991	MZFC 36
*Imantodes gemmistratus* (Cope, 1861)	4	LC	Pr	L (6)	3	1951	AMNH R-71361
*Leptodeira maculata* (Hallowell, 1861)	4,6	LC	Pr	L (7)	1	1965	CNAR 1102
*Leptodeira septentrionalis* (Kennicott, 1859)	4	LC	NL	L (8)	4	1992	Camarillo-Rangel and Smith 1992
*Leptodeira splendida* Günther, 1895	4	LC	NL	H (14)	1	1976	CNAR 3770
*Rhadinaea hesperia* Bailey, 1940	4	LC	Pr	M (10)	1	1973	ENCB 7829
*Rhadinaea laureata* (Günther, 1868)	1,2,3	LC	NL	M (12)	1	1952	KUNHM 39966
*Rhadinaea taeniata* (Peters, 1863)	1,2	LC	NL	M (13)	1	1979	CNAR 3543
**FAMILY ELAPIDAE (3)**							
*Micrurus browni* Schmidt & Smith, 1943	1,2	LC	Pr	L (8)	3	1954	KUNHM 50701
*Micrurus laticollaris* Peters, 1870	4	LC	Pr	H (14)	1	1986	ENCB 12924
*Micrurus tener* Baird & Girard, 1953	1,4	LC	NL	M (11)	2	1943	ENCB 2204
**FAMILY LEPTOTYPHLOPIDAE (2)**							
*Epictia bakewelli* (Oliver, 1937)	4	NE	NL	NE	1	1985	Camarillo-Rangel et al. 1985
*Rena maxima* (Loveridge, 1932)	4	LC	NL	M (11)	1	1960	KUNHM 67639
**FAMILY NATRICIDAE (7)**							
*Storeria storerioides* (Cope, 1866)	1,2,3	LC	NL	M (11)	1	1938	UIMNH 18771
*Thamnophis cyrtopsis* (Kennicott, 1860)	1,2,3,4,6	LC	A	L (7)	4	1892	USNM 19003
*Thamnophis eques* (Reuss, 1834)	1,2,3,4,6	LC	A	L (8)	2	1904	USNM 46599
*Thamnophis melanogaster* (Wiegmann, 1830)	1,2,3,6	EN	A	H (15)	1	1888	Dugès 1888
*Thamnophis pulchrilatus* (Cope, 1885)	1,2,3,4	LC	NL	H (15)	1	1888	Dugès 1888
*Thamnophis scalaris* Cope, 1861	1,2,3,5	LC	A	H (14)	1	1888	Dugès 1888
*Thamnohis scaliger* (Jan, 1863)	1,2,3,5,6	VU	A	H (15)	1	1939	UMMZ 85367
**FAMILY TYPHLOPIDAE (1)**							
*Indotyphlops braminus* (Daudin, 1803)	**IN**	**IN**	**IN**	**IN**	**IN**	1997	CNAR 11307
**FAMILY VIPERIDAE (10)**							
*Crotalus aquilus* Klauber, 1952	1,2,3,4,6	LC	Pr	H (16)	1	1982	CNAR 4246
*Crotalus atrox* Baird & Girard, 1853	5	LC	Pr	L (9)	2	2004	Matias-Ferrer and Murillo 2004b
*Crotalus culminatus* Klauber, 1952	4	NE	NL	H (15)	1	1888	Dugès 1888
*Crotalus molossus* Baird & Girard, 1853	1,2,3,6	LC	Pr	L (8)	2	1888	Dugès 1888
*Crotalus polystictus* (Cope, 1865)	1,2,3,4	LC	Pr	H (16)	1	1888	Dugès 1888
*Crotalus ravus* Cope, 1865	1,2,3,4,6	LC	A	H (14)	1	1938	UIMNH 19186
*Crotalus scutulatus* (Kennicott, 1861)	5	LC	Pr	M (11)	2	1967	ENCB 3853
*Crotalus tlaloci* Bryson, Linkem, Dorcas, Lathrop, Jones, Alvarado-Días, Grünwald & Murphy, 2014	1,2,3,4	NE	NL	H (16)	1	2014	Bryson et al. 2014
*Crotalus transversus* Taylor, 1944	2,3	LC	P	H (17)	1	1973	KUNHM 159362
*Crotalus triseriatus* (Wagler, 1830)	1,2,3,4,6	LC	NL	H (16)	1	1940	MVZ 36745
**ORDER TESTUDINES**							
**EMYDIDAE (1)**							
*Trachemys venusta* (Gray, 1855)	4	NE	NL	M (13)	3	1939	MCZ R-45542
**FAMILY GEOEMYDIDAE (1)**							
*Rhinoclemmys rubida* (Cope, 1870)	4	NT	Pr	H (14)	1	1983	Camarillo-Rangel 1983
**FAMILY KINOSTERNIDAE (2)**							
*Kinosternon hirtipes* (Wagler, 1830)	1,4,5,6	LC	Pr	M (10)	2	1888	Dugès 1888
*Kinosternon integrum* LeConte, 1854	4	LC	Pr	M (11)	1	1888	Dugès 1888

### Habitat types

In the State of Mexico, the percentage of herpetofaunal species found in the Oak (51.7%), Pine-oak (55.8%), Pine (44.9%), and Tropical Deciduous Forest (51.7%) vegetation types are relatively equal (Table 1). However, the Grassland (29.9%) and Scrubland (23.8%) vegetation types have relatively fewer species. This pattern of the observed percentage of species in each habitat type is the same for amphibians and reptiles individually in the Oak, Pine-oak, and Pine Forests; and in the Scrubland. However, the Tropical Deciduous Forest contains a higher percentage of reptiles (80.3%) than for amphibians (19.7%), which might be due to the dry conditions of this vegetation type. The percentage of species found in the Grassland is the same for amphibians as for reptiles (50.0% for both), perhaps due to the high altitude grasslands that intermingle with Pine Forest in the State of Mexico, and these grasslands often traverse streams which host important populations of hylids, ranids, ambystomatids, anguids, phrynosomatids, colubrids, and vipers in the State of Mexico.

**Table 2.** List of amphibian and reptile species that potentially occur in the State of Mexico.

	Region in the State of Mexico where it likely occurs
**CLASS AMPHIBIA**	
**ORDER ANURA**	
**Family Craugastoridae**	
*Craugastor rhodopis* (Cope, 1867)	southern
**Family Hylidae**	
*Scinax staufferi* (Cope, 1865)	southern
**CLASS REPTILIA**	
**ORDER SQUAMATA**	
**SUBORDER AMPHISBAENIA**	
**Family Bipedidae**	
*Bipes canaliculatus* Latreille, 1801	extreme southwestern
**SUBORDER LACERTILIA**	
**Family Anguidae**	
*Gerrhonotus ophiourus* Cope, 1867	eastern and southern
**Family Eublepharidae**	
*Coleonyx elegans* Gray, 1845	extreme southwestern
**Family Phrynosomatidae**	
*Phrynosoma asio* Cope, 1864	extreme southwestern
*Sceloporus minor* Cope, 1885	northern
*Sceloporus siniferus* Cope, 1870	extreme southwestern
*Sceloporus utiformis* Cope, 1864	extreme southwestern
**Family Phyllodactylidae**	
*Phyllodactylus bordai* Taylor, 1942	extreme southwestern
*Phyllodactylus tuberculosus* Wiegmann, 1834	extreme southwestern
**Family Teiidae**	
*Holcosus sinister* (Wiegmann, 1834)	extreme southwestern
**SUBORDER SERPENTES**	
**Family Colubridae**	
*Ficimia publia* (Cope, 1866)	extreme southwestern
*Lampropeltis ruthveni* Blanchard, 1920	northern
*Mastigodryas melanolomus* (Cope, 1868)	extreme southwestern
*Sonora michoacanensi* (Dugès, 1884)	western and southwestern
**Family Dipsadidae**	
*Pseudoleptodeira latifasciata* (Günther, 1894)	extreme southwestern
*Tropidodipsas zweifeli* (Liner & Wilson, 1970)	extreme southwestern
**Family Loxocemidae**	
*Loxocemus bicolor* Cope, 1861	extreme southwestern
**Family Viperidae**	
*Agkistrodon bilineatus* Günther, 1863	extreme southwestern
**ORDER TESTUDINES**	
**Family Kinosternidae**	
*Kinosternon scorpioides* (Linnaeus, 1766)	western and southwestern

### Conservation status

**Figure 8.**
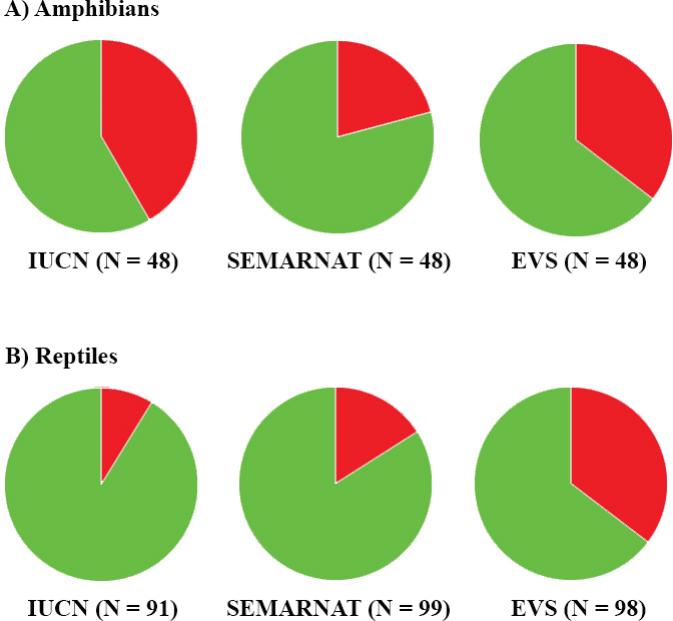
Proportion of **A**) amphibians and **B**) reptiles listed in protected categories on the IUCN Red List, SEMARNAT, and high EVS for the State of Mexico. Green is proportion in Data Deficient and Least Concern (IUCN); Not Listed and Subject to Special Protection (we regarded the category of Subject to Special Protection in SEMARNAT equivalent to Least Concern in IUCN) (SEMARNAT); or low or medium EVS. Red is percentage in protected categories or high EVS. N is the number of species assessed.

Of the amphibian and reptile species in the State of Mexico, 20.1% are IUCN listed (i.e., Vulnerable, Near Threatened, or Endangered), 18.4% are placed in a protected category by SEMARNAT (excluding NL and Pr, this last category is equivalent to the LC category of IUCN), and 34.9% are categorized as high risk by the EVS (Table 3; Fig. 8). For amphibians, 41.7% are IUCN listed, 20.8% are protected by SEMARNAT, and 33.3% are at high risk according to the EVS (Table 3; Fig. 8). For reptiles, 8.8% are listed by the IUCN, 17.2% are protected by SEMARNAT, and 35.7% are at high risk according to the EVS (Table 3; Fig. 8). These results suggest that many amphibians found in the State of Mexico are at risk and of relatively high conservation concern at both the global and national scale. However, the reptiles found in the State of Mexico are less at risk according to the global and national assessments of the IUCN and SEMARNAT, respectively; but the EVS suggests they may be at higher risk than the IUCN and SEMARNAT assessments suggest. Based on our review of the conservation statuses of the herpetofauna found in the State of Mexico, we have identified several families that include species of particular conservation concern. These families include Craugastoridae, Eleutherodactylidae, Ambystomatidae, Plethodontidae, Helodermatidae, Iguanidae, Phrynosomatidae, Colubridae, Natricidae, and Viperidae (Table 3). Because the conservation statuses we reviewed are developed and applied at a species wide level, we believe that the conservation status of specific taxa in the State of Mexico may not be accurately reflected by these measures. Additional state level assessments are needed, especially for species in the families we have identified as being at a particularly high level of risk.

**Table 3.** Summary of native species present in the State of Mexico by family, order or suborder, and class. Status summary indicates the number of species found in each IUCN conservation status in the order DD, LC, VU, NT, EN, CR (see Table 1 for abbreviations; in some cases species have not been assigned a status by the IUCN and therefore these may not add up to the total number of species in a taxon). Mean EVS is the mean Environmental Vulnerability Score; scores ≥ 14 are considered high vulnerability (Wilson et al. 2013a, b) and conservation status in Mexico according to SEMARNAT (2019) in the order NL, Pr, A, P (see Table 1 for abbreviations).

Scientific name	Genera	Species	IUCN	x̅ EVS	SEMARNAT
			DD, LC, VU, NT, EN, CR		NL, Pr, A, P
**CLASS AMPHIBIA**					
**ORDER ANURA**	**15**	**32**	**1,24,2,1,3,1**	**10.3**	**20,9,2,1**
Bufonidae	3	5	0,4,0,0,1,0	10.4	5,0,0,0
Craugastoridae	1	4	0,2,1,0,1,0	11.25	4,0,0,0
Eleutherodactylidae	1	4	1,2,1,0,0,0	14.3	2,2,0,0
Hylidae	5	9	0,8,0,0,1,0	9.3	6,2,1,0
Leptodactylidae	1	1	0,1,0,0,0,0	6	0,1,0,0
Microhylidae	1	1	0,1,0,0,0,0	4	0,1,0,0
Phyllomedusidae	1	1	0,1,0,0,0,0	13	0,1,0,0
Ranidae	1	6	0,4,0,1,0,1	11.2	2,2,1,1
Scaphiopodidae	1	1	0,1,0,0,0,0	3	1,0,0,0
**ORDER CAUDATA**	**5**	**16**	**1,2,2,1,5,5**	**14.8**	**2,7,7,0**
Ambystomatidae	1	8	1,1,0,0,2,4	13.5	0,5,3,0
Plethodontidae	4	8	0,1,2,1,3,1	16.1	2,2,4,0
**SUBTOTAL**	**20**	**48**	**2,26,4,2,8,6**	**11.8**	**22,16,9,1**
**CLASS REPTILIA**					
**ORDER SQUAMATA**	**40**	**95**	**2,79,3,0,4,0**	**11.5**	**53,25,15,2**
**SUBORDER LACERTILIA**	**13**	**39**	**0,32,2,0,3,0**	**12.4**	**25,9,4,1**
Anguidae	3	5	0,2,0,0,3,0	13.2	1,2,1,1
Dactyloidae	1	1	0,1,0,0,0,0	13	1,0,0,0
Helodermatidae	1	1	0,1,0,0,0,0	11	0,0,1,0
Iguanidae	1	1	0,0,0,0,0,0	15	0,0,1,0
Phrynosomatidae	3	19	0,18,1,0,0,0	12.4	16,2,1,0
Phyllodactylidae	1	1	0,1,0,0,0,0	15	1,0,0,0
Scincidae	2	6	0,4,1,0,0,0	12	3,3,0,0
Teiidae	1	5	0,5,0,0,0,0	11.2	3,2,0,0
**SUBORDER SERPENTES**	**27**	**56**	**2,47,1,0,1,0**	**11**	**28,16,11,1**
Boidae	1	1	0,0,0,0,0,0	15	1,0,0,0
Colubridae	13	21	0,20,0,0,0,0	10	12,4,5,0
Dipsadidae	7	12	2,10,0,0,0,0	9.8	7,5,0,0
Elapidae	1	3	0,3,0,0,0,0	11	1,2,0,0
Leptotyphlopidae	2	2	0,1,0,0,0,0	11	2,0,0,0
Natricidae	2	7	0,5,1,0,1,0	11.5	2,0,5,0
Viperidae	1	10	0,8,0,0,0,0	13.8	3,5,1,1
**ORDER TESTUDINES**	**3**	**4**	**0,2,0,1,0,0**	**12**	**1,3,0,0**
Emydidae	1	1	0,0,0,0,0,0	13	1,0,0,0
Geoemydidae	1	1	0,0,0,1,0,0	14	0,1,0,0
Kinosternidae	1	2	0,2,0,0,0,0	10.5	0,2,0,0
**SUBTOTAL**	**43**	**99**	**2,81,3,1,4,0**	**11.6**	**54,28,15,2**
**TOTAL**	**63**	**147**	**4107,7,3,12,6**	**11.7**	**76,44,24,3**

We summarized the conservation status of amphibian and reptile taxa in each vegetation type found in the State of Mexico to determine the vegetation types that support species of particular conservation concern (Table 1). For IUCN listings, 43.3% of amphibian species in the Oak Forest are listed in a protected category; 48.5% in the Pine-oak Forest; 50.0% in the Pine Forest; 13.3% in the Tropical Deciduous Forest; 59.1% in the Grassland; and 16.7% in the Scrubland. For SEMARNAT listings of amphibian species, 30.0% in the Oak Forest are listed in a protected category; 30.3% in the Pine-oak Forest; 34.6% in the Pine Forest; 0% in the Tropical Deciduous Forest; 36.4% in the Grassland; and 16.7% in the Scrubland. For EVS, 40.0% of amphibian species in the Oak Forest of the State of Mexico were in the high category, 45.5% in the Pine-oak Forest, 42.3% in the Pine Forest, 0% in the Tropical Deciduous Forest, 54.5% in the Grassland, and 25.0% in the Scrubland. For IUCN listings, 8.9% of reptile species in the Oak Forest are listed in a protected category; 12.5% in the Pine-oak Forest; 12.5% in the Pine Forest; 3.8% in the Tropical Deciduous Forest; 9.1% in the Grassland; and 8.7% in the Scrubland. For SEMARNAT listings of reptile species, 22.2% in the Oak Forest are listed in a protected category; 25.0% in the Pine-oak Forest; 25.6% in the Pine Forest; 15.0% in the Tropical Deciduous Forest; 18.2% in the Grassland; and 34.8% in the Scrubland. For EVS, 42.2% of reptile species in the Oak Forest of the State of Mexico were in the high category, 45.8% in the Pine-oak Forest, 53.8% in the Pine Forest, 35.0% in the Tropical Deciduous Forest, 22.7% in the Grassland, and 34.8% in the Scrubland. Given the apparent importance of forested habitats in terms of protected amphibian and reptile species in the State of Mexico, efforts to maintain or expand such habitats, perhaps by reforestation, is a management strategy that needs to be considered. Indeed, Sánchez-Jasso et al. (2013) found that reforested woodlands in the State of Mexico supported a relatively high richness of vertebrates.

### Comparison with neighboring states

Overall, the State of Mexico shares the most species (76.9%) with Michoacán (Table 4). The State of Mexico also shares the most amphibian species with Michoacán (72.9%), including 87.5% of its anuran species, and 43.8% of its salamander species. These two states are especially important for salamanders in the family Ambystomatidae and contribute 11 of the 14 species of the regional pool, only lacking *A. mexicanum* (endemic to Mexico City), *A. taylori* (endemic to Puebla), and *A. subsalsum*. For reptiles, the State of Mexico shares 78.8% of its reptile species with Michoacán. The similarity between these two states is due to the long border between them (241 km, INEGI 2018) and the fact that the larger Michoacán contains essentially all of the vegetation types present in the State of Mexico. In contrast, the state that shares the second highest number of species with the State of Mexico is the small state of Morelos. Morelos, along with the State of Mexico and Mexico City, share parts of the Corredor Biológico Chichinautzin, which includes the Lagunas de Zempoala National Park, that hosts a unique assortment of amphibians and reptiles. Moreover, Morelos shares part of the Tropical Deciduous Forest with the southern part of the State of Mexico. Puebla and Guerrero also share a large number of species with the State of Mexico. Hidalgo, Querétaro, and Mexico City share fewer amphibian and reptile species with the State of Mexico. Hidalgo and Querétaro are states whose dominant species are from the Mexican Altiplano and the Sierra Madre Oriental, whereas the dominant species for the State of Mexico are a combination of species of the Eje Neovolcánico and the Sierra Madre del Sur. The lower number of shared species among these states may also reflect the inherent species richness of the shared habitat types. In addition, the border of Querétaro with the State of Mexico is quite short (95.3 km, INEGI 2018), and although the border of Hidalgo with the State of Mexico is the longest of the other neighboring states (422.3 km, INEGI 2018), most of this border is confined to the subprovince of Llanuras and Sierras de Querétaro e Hidalgo, with a sole contribution of species typical of the Mexican Altiplano. On the other hand, although Mexico City is nearly surrounded by the State of Mexico, its small size (1,485 km^2^) along with its large urbanized area, results in a small number of species of amphibians and reptiles (63: Lemos-Espinal and Smith, in press), which also results in an equally small number of species shared between Mexico City and the State of Mexico (59). However, 93.7% of the total number of species recorded for Mexico City is shared with the State of Mexico.

**Table 4.** Summary of the numbers of species shared between the State of Mexico and neighboring Mexican states (not including introduced species). The percent of the State of Mexico species shared by a neighboring state are given in parentheses. – indicates either the State of Mexico or the neighboring state has no species in the taxonomic group, or none of that specific taxon is shared between the states, thus no value for shared species is provided.

Taxon	State of Mexico	Michoacán	Morelos	Puebla	Guerrero	Hidalgo	Querétaro	Mexico City
**CLASS AMPHIBIA**	**48**	**35 (72.9)**	**33 (68.8)**	**27 (56.3)**	**26 (55.3)**	**20 (41.7)**	**16 (33.3)**	**16 (33.3)**
**ORDER ANURA**	**32**	**28 (87.5)**	**26 (81.3)**	**23 (71.9)**	**23 (71.9)**	**16 (50.0)**	**13 (40.6)**	**8 (25.0)**
Bufonidae	5	5 (100)	5 (100)	5 (100)	4 (80.0)	3 (60.0)	3 (60.0)	1 (20.0)
Craugastoridae	4	3 (75.0)	4 (100)	3 (75.0)	4 (100)	1 (25.0)	1 (25.0)	1 (25.0)
Eleutherodactylidae	4	3 (75.0)	3 (75.0)	1 (25.0)	2 (50.0)	1 (25.0)	1 (25.0)	–
Hylidae	9	8 (88.9)	7 (77.8)	7 (77.8)	7 (77.8)	5 (55.6)	3 (33.3)	3 (33.3)
Leptodactylidae	1	1 (100)	0	1 (100)	1 (100)	1 (100)	–	–
Microhylidae	1	1 (100)	1 (100)	1 (100)	1 (100)	1 (100)	1 (100)	–
Phyllomedusidae	1	1 (100)	1 (100)	1 (100)	1 (100)	–	–	–
Ranidae	6	5 (83.3)	4 (66.7)	3 (50.0)	2 (33.3)	3 (50.0)	3 (50.0)	2 (33.3)
Scaphiopodidae	1	1 (100)	1 (100)	1 (100)	1 (100)	1 (100)	1 (100)	1 (100)
**ORDER CAUDATA**	**16**	**7 (43.8)**	**7 (43.8)**	**4 (25.0)**	**3 (18.8)**	**4 (25.0)**	**3 (18.8))**	**8 (50.0)**
Ambystomatidae	8	4 (50.0)	1 (12.5)	1 (12.5)	1 (12.5)	1 (12.5)	1 (12.5)	2 (25.0)
Plethodontidae	8	3 (37.5)	6 (75.0)	3 (37.5)	2 (25.0)	3 (37.5)	2 (25.0)	6 (75.0)
**CLASS REPTILIA**	**99**	**78 (78.8)**	**73 (71.8)**	**71 (71.7)**	**65 (65.7)**	**47 (47.5)**	**45 (45.5)**	**43 (43.4)**
**ORDER SQUAMATA**	**95**	**75 (78.9)**	**71 (74.7)**	**69 (72.6)**	**63 (66.3)**	**44 (46.3)**	**43 (45.3)**	**41 (43.2)**
**SUBORDER LACERTILIA**	**39**	**28 (71.8)**	**29 (74.4)**	**26 (66.7)**	**26 (66.7)**	**12 (30.8)**	**10 (25.6)**	**14 (35.9)**
Anguidae	5	3 (60.0)	4 (80.0)	2 (40.0)	3 (60.0)	1 (20.0)	1 (20.0)	1 (20.0)
Dactyloidae	1	1 (100)	1 (100)	–	1 (100)	–	–	–
Helodermatidae	1	1 (100)	1 (100)	1 (100)	1 (100)	–	–	–
Iguanidae	1	1 (100)	1 (100)	1 (100)	1 (100)	–	–	–
Phrynosomatidae	19	12 (63.2)	14 (73.7)	15 (78.9)	12 (63.2)	9 (47.4)	7 (36.8)	10 (52.6)
Phyllodactylidae	1	1 (100)	–	–	1 (100)	–	–	–
Scincidae	6	4 (66.7)	4 (66.7)	4 (66.7)	3 (50.0)	1 (16.7)	1 (16.7)	2 (33.3)
Teiidae	5	5 (100)	4 (80.0)	3 (60.0)	4 (80.0)	1 (20.0)	1 (20.0)	1 (20.0)
**SUBORDER SERPENTES**	**56**	**47 (83.9)**	**42 (75.0)**	**43 (76.8)**	**37 (66.1)**	**32 (57.1)**	**33 (58.9)**	**27 (48.2)**
Boidae	1	1 (100)	1 (100)	1 (100)	1 (100)	–	–	–
Colubridae	21	19 (90.5)	19 (90.5)	20 (95.2)	18 (85.7)	13 (61.9)	14 (66.7)	9 (42.9)
Dipsadidae	12	11 (91.7)	8 (66.7)	7 (58.3)	8 (66.6)	5 (41.7)	4 (33.3)	4 (33.3)
Elapidae	3	2 (66.7)	2 (66.7)	2 (66.7)	2 (66.6)	1 (33.3)	1 (33.3)	1 (33.3)
Leptotyphlopidae	2	2 (100)	1 (50.0)	1 (50.0)	2 (100)	1 (50.0)	1 (50.0)	–
Natricidae	7	7 (100)	4 (57.1)	6 (85.7)	3 (42.9)	6 (85.7)	6 (85.7)	7 (100)
Viperidae	10	5 (50.0)	7 (70.0)	6 (60.0)	3 (30.0)	6 (60.0)	7 (70.0)	6 (60.0)
**ORDER TESTUDINES**	**4**	**3 (75.0)**	**2 (50.0)**	**2 (50.0)**	**2 (50.0)**	**3 (75.0)**	**2 (50.0)**	**2 (50.0)**
Emydidae	1	–	–	1 (100)	–	1 (100)	–	–
Geoemydidae	1	1 (100)	–	–	1 (100)	–	–	–
Kinosternidae	2	2 (100)	2 (100)	1 (50.0)	1 (50.0)	2 (100)	2 (100)	2 (100)
**TOTAL**	**147**	**113 (76.9)**	**106 (72.1)**	**98 (66.7)**	**91 (61.9)**	**67 (45.6)**	**61 (41.5)**	**59 (40.1)**

## Appendix 1

Museum collections included in the VertNet.org database records of the State of Mexico amphibians and reptiles that house specimens of the first record of a species in the State of Mexico.

AMNHCollection of Herpetology, Herpetology Department, American Museum of Natural History;

CNARColección Nacional de Anfibios y Reptiles, Instituto de Biología, Universidad Nacional Autónoma de México;

ENCBEscuela Nacional de Ciencias Biológicas, Instituto Politécnico Nacional;

FMNHDivision of Amphibians and Reptiles, Field Museum of Natural History;

MCZCollection of Herpetology, Museum of Comparative Zoology, Harvard University Cambridge;

KUNHM Museum of Natural History, Division of Herpetology, University of Kansas;

MVZMuseum of Vertebrate Zoology at Berkeley, Herpetological Collection;

MZFCMuseo de Zoología Alfonso L. Herrera, Facultad de Ciencias, UNAM. Colección Herpetológica;

NHMNatural History Museum, London, Zoological Collection;

TCWCCollection of Herpetology, Texas Cooperative Wildlife Collection, Texas A&M University;

UCMCollection of Herpetology, University of Colorado Museum;

UIMNHUniversity of Illinois Museum of Natural History Amphibian and Reptile Collection;

UMMZCollection of Herpetology, Museum of Zoology, University of Michigan Ann Arbor;

USNMCollection of Herpetology, Department of Vertebrate Zoology, National Museum of Natural History, Smithsonian Institution;

UTAMM Merriam Museum, University of Texas Arlington;

UTEPCollection of Herpetology, Laboratory of Environmental Biology, Biological Science Department, University of Texas – El Paso.
